#  Impact of Healthcare-Associated Infections on Length of Stay: A Study in 68 Hospitals in China

**DOI:** 10.1155/2019/2590563

**Published:** 2019-04-18

**Authors:** Huixue Jia, Liuyi Li, Weiguang Li, Tieying Hou, Hongqiu Ma, Yun Yang, Anhua Wu, Yunxi Liu, Jianguo Wen, Huai Yang, Xiaoli Luo, Yawei Xing, Weihong Zhang, Yinghong Wu, Lili Ding, Weiping Liu, Ling Lin, Ying Li, Meilian Chen

**Affiliations:** ^1^Peking University First Hospital, Beijing 100034, China; ^2^Shandong Provincial Hospital, Jinan 250021, China; ^3^Guangdong General Hospital, Guangzhou 510008, China; ^4^The First Affiliated Hospital of Anhui Medical University, Hefei 230022, China; ^5^The First Hospital, Shanxi Medical University, Taiyuan 030001, China; ^6^Xiangya Hospital, Central South University, Changsha 410008, China; ^7^General Hospital of PLA, Beijing 100853, China; ^8^The First Affiliated Hospital of Zhengzhou University, Zhengzhou 450052, China; ^9^Guizhou Provincial People's Hospital, Guiyang 550002, China; ^10^Jiangxi Provincial Children's Hospital, Nanchang 330006, China; ^11^Fourth Hospital of Hebei Medical University, Shijiazhuang 050019, China; ^12^Jiangsu Province Hospital, Nanjing 210029, China; ^13^Peking University People's Hospital, Beijing 100044, China; ^14^The First Affiliated Hospital of Xinjiang Medical University, Urumqi 830054, China; ^15^Inner Mongolia People's Hospital, Hohhot 010017, China; ^16^Heilongjiang Provincial Center for Disease Control and Prevention, Harbin 150030, China

## Abstract

Healthcare-associated infections (HAIs) not only bring additional medical cost to the patients but also prolong the length of stay (LOS). 2119 HAI case-patients and 2119 matched control-patients were identified in 68 hospitals in 14 primary sampling provinces of 7 major regions of China. The HAI caused an increase in stay of 10.4 days. The LOS due to HAI increased from 9.7 to 10.9 days in different levels of hospitals. There was no statistically significant difference in the increased LOS between different hospital levels. The increased LOS due to HAI in different regions was 8.2 to 12.6 days. Comparing between regions, we found that the increased LOS due to HAI in South China is longer than other regions except the Northeast. The gastrointestinal infection (GI) caused the shortest extra LOS of 6.7 days while the BSI caused the longest extra LOS of 12.8 days. The increased LOS for GI was significantly shorter than that of other sites. Among 2119 case-patients, the non-multidrug-resistant pathogens were detected in 365 cases. The average increased LOS due to these bacterial infections was 12.2 days.* E. coli* infection caused significantly shorter LOS. The studied MDROs, namely, MRSA, VRE, ESBLs-*E. coli*, ESBLs-KP, CR-*E. coli*, CR-KP, CR-AB, and CR-PA were detected in 381 cases (18.0%). The average increased LOS due to these MDRO infections was 14 days. Comparing between different MDRO infections, we found that the increased LOS due to HAI caused by CR-PA (26.5 days) is longer than other MDRO infections (shorter than 19.8 days).

## 1. Introduction

Healthcare-associated infections (HAIs) not only threaten the patients' health and life but also bring additional economic burden to the patients and healthcare system including direct economic loss and prolonged hospitalization. Total hospital length of stay (LOS) is known to be prolonged by the occurrence of HAI.

An increased length of stay of 5 days due to HAIs in the ICU was estimated in a study of France [1]. The excess of days of hospitalization for infected patients in ICU was 7.7 days in another study [2].

For different infection sites, the extra LOS was 27.1 days, 22.2 days, and 19.2 days for CLABSI, VAP, and CAUTI, respectively, in adult and pediatric ICUs [3]. The mean LOS attributable to CLABSIs was 19 days in another study [4]. The extra length of stay was 3.48 days for BSI, 3.59 days for UTI, 7.23 days for SSI, and 11.52 days for VAP in medical-surgical ICU [2].

Most studies show that multidrug-resistant organism (MDRO) infections cause extra LOS of 2.0–12.7 days compared with those caused by susceptible strains [5–11].

However, many studies on the increased LOS due to HAIs had poor homogeneity and comparability, because most studies were limited to infections caused by a single site or a single organisms, and the characteristics of patients in the studies were different [12–14].

At present, many studies have reported the effect of HAIs on the LOS, but the LOS varies according to the site of infection, infection of pathogens, and different hospital levels. Previous studies have not systematically analyzed the above related factors, and there is a lack of large scale and large sample research.

Our study aimed to evaluate the impact of HAIs on LOS from different hospital levels, different regions, different infection sites, different pathogens, and different MDROs systematically in China.

## 2. Materials and Methods

### 2.1. Sampling Methods

This survey was conducted in 68 hospitals in 14 primary sampling provinces (Shandong, Guangdong, Anhui, Shanxi, Hunan, Henan, Guizhou, Jiangxi, Hebei, Jiangsu, Beijing, Xinjiang, Inner Mongolia, and Heilongjiang) of 7 major regions of China (Northeast, North, Central, East, South, Northwest, and Southwest). Each province had at least one provincial or ministerial level general hospital, one prefectural or municipal level general hospital, and/or one district or county level general hospital.

### 2.2. Patients

From January 1 to December 31, 2015, 50 patients with HAIs were randomly selected in one hospital including 10 lower respiratory tract infections [(LRTI) including ventilator-associated pneumonia (VAP)], 10 urinary tract infections [(UTI) including catheter associated urinary tract infections (CAUTI)], 10 gastrointestinal infections [(GI) including infectious diarrhea, gastrointestinal infection, and antimicrobial associated diarrhea], 10 surgical site infections (SSI), and 10 blood stream infections [(BSI) including central catheter associated blood stream infection (CLABSI)]. If the infections in one site were less than 10 cases, all cases were investigated. One control patient was selected for one case-patient. The matching principle included the same sex, age difference of less than 5 years, the same or similar first diagnosis (the main diseases in the hospital), and the same or similar surgical procedure if it applies. The control-patients should have stayed in the hospital more than 48 hours and have no incurred healthcare-associated infections. If the HAI subject is 2 to 5 years old with the age difference being less than 1 year or if the HAI subject is less than 2 years old, then the matching age is the same. The case-patients were excluded if they had the following conditions: (1) patients with 2 or more HAIs; (2) patients in geriatric ward or intensive care unit or with long-term coma (> 1 month), long stay in hospital (> 3 months) due to vegetative or other noninfectious causes (such as medical dispute); (3) patients with infection upon admission; or (4) patients with no matched controls.

### 2.3. Pathogens

This study mainly monitored multidrug-resistant or non-multidrug-resistant* Staphylococcus aureus* (SA),* Staphylococcus epidermidis* (SE),* Enterococcus* (EC),* Escherichia coli* (*E. coli*),* Klebsiella pneumonia* (KP),* Acinetobacter baumannii* (AB), and* Pseudomonas aeruginosa* (PA). MDRO species were methicillin-resistant* Staphylococcus aureus* (MRSA), methicillin-resistant* Staphylococcus epidermidis* (MRSE), vancomycin resistant* Enterococcus* (VRE), extended-spectrum *β*-lactamases producing (ESBLs)* Escherichia coli* and* Klebsiella pneumoniae*, carbapenem resistant* Escherichia coli* (CR-*E. coli*) and* Klebsiella pneumoniae* (CR-KP), carbapenem resistant* Acinetobacter baumannii* (CR-AB), and carbapenem resistant* Pseudomonas aeruginosa* (CR-PA).

### 2.4. Definition

HAIs were defined using the Chinese criteria [15].

### 2.5. Data Analysis

The patients with HAIs were assumed as the case group and patients without any HAIs as the control group. The total LOS was evaluated for the case. The descriptive statistics and frequency distribution such as mean ($\overset{-}{x}$), standard deviation (SD), and percentage were used.

The differences were analyzed by matched T tests. Due to the skewed distribution of the LOS of the patients, the matched T test was made after logarithmic conversion. All of the statistical analyses were two sided, and P < 0.05 was considered significant. Also, SPSS, version 18 was used for data analysis.

## 3. Results

A total of 49540 HAIs occurred in the 68 surveyed hospitals in 2015, and the number of HAIs at five surveyed sites was 38858. 2119 HAI case-patients and 2119 matched control-patients were identified in this study. The median age of the case group was 59 years, the quartile interval was 29 years, the youngest was 0 years, and the oldest was 98 years old. The median age of the control group was 59 years, the quartile spacing was 28 years, the youngest was 0 years, and the maximum was 96 years. The ratios of male to female were the same in two groups. 55.11% (1170 cases) were male and 44.89% (953 cases) were female.

The average total LOS of the case group was 21.3±1.9 days and that of the control group was 10.9±1.9 days. The difference between the case and control group was statistically significant (t = 45.13, P < 0.01). The HAI caused an increase in average stay of 10.4 days.

The LOS due to HAI increased, 9.7-10.9 days, in different levels of hospitals: 9.7 days in district or county hospitals, 10.4 days in prefectural or municipal hospitals, and 10.9 days in provincial or ministerial hospitals, respectively. The LOS in the case groups was significantly higher than that of the control groups (P < 0.01) for each level hospital. There was no significant difference in the increased LOS between different hospital levels (P > 0.05) (Table 1).

The increased LOS due to HAI in different regions was 8.2-12.6 days. The increased LOS in Northwest China is the shortest while that of South China is the longest. The LOS in the case group was significantly longer than that of the control group in each region (P < 0.01). Comparing between regions, we discovered that the increased LOS due to HAI in South China is obviously longer than other regions except the Northeast. The increased LOS due to HAI in Northwest China is obviously shorter than other regions except Central and Southwest China where the difference was statistically significant [(P < 0.05) (Table 2)].

The increased LOS due to HAI was different in different infection sites which was 6.7-12.8 days. The GI caused the shortest increase in stay of 6.7 days while the BSI caused the longest increase in stay of 12.8 days. The LOS of the patients with different infection sites was significantly longer than that of those corresponding controls (P < 0.01). The increased LOS of GI was significantly shorter than that of other sites (P < 0.05) but there was no significant difference among LRTI, UTI, SSI, and BSI [(P > 0.05) (Table 3)].

Among 2119 case-patients, 365 cases detected the studied non-multidrug-resistant pathogens. The average increased LOS due to these bacterial infections was 12.2 days, among which the LOS of KP infection was the most prolonged with 15.5 days followed by EC and SA infection. The LOS of the patients with different pathogen infections was longer than that of the control groups (P < 0.01). Compared with SA and KP infection,* E. coli* infection caused significantly shorter LOS (P < 0.05); there was no significant difference among other pathogen infections [(P > 0.05) (Table 4)].

Among 2119 case-patients, the studied MDROs were detected in 381 cases. The average increased LOS due to these MDRO infections was 14 days, among which the LOS of CR-PA infection was the most prolonged which was 26.5 days. The increased shortest LOS was 9.7 days due to MRSE infection. The LOS of the patients with different MDRO infections was longer than that of the control groups (P < 0.01). Comparing between different MDRO infections, we found that the increased LOS due to HAI caused by CR-PA is obviously longer than other MDRO infections except VRE and CR-*E. coli* infections (P < 0.05); there was no significant difference among other MDRO infections [(P > 0.05) (Table 5)].

## 4. Discussion

Healthcare-associated infections (HAIs) affect millions of patients worldwide. HAIs are associated with increased hospital length of stay (LOS), thus increasing the healthcare cost [16], which not only burdens medical resources but also increases patients' suffering and even causes medical disputes.

This study found that the increased LOS of HAI was about twice as long as those of the noninfected patients, with an average prolongation of 10.4 days which was close to the results of Sun Jihua [17] and Zhou Chunlian [18].

The increased LOS due to HAI was not related to hospital level but there were regional differences. The clear understanding of the underlining reasons is still lacking.

In this study, the effects of different infection sites on LOS were analyzed. Among them, BSI prolongs hospital stay for 12.8 days. In a multicenter study, involving 69 tertiary-care ICUs of 37 cities in 11 countries, the extra LOS due to CVC-BSI was 9.8 days [19]. In a Brazil study, the increased LOS attributable to BSI was 23.63 days [20]. Through these studies, we can see that BSI can cause longer days of hospitalization. In addition, the extra LOS due to GI was the shortest compared with other infection sites which may be related to the easy treatment of GI and less influence on patients with underlying diseases.

The average extra LOS was 12.2 days for non-drug-resistant pathogens and 14 days for MDRO infection. The average extra LOS of MDRO infection was 2 days longer than that of nonresistant bacteria. Among the non-drug-resistant bacteria,* E. coli* had the shortest extra LOS (9.8 days), but drug-resistant* E. coli* prolonged the length of stay more than 11.7 days. The increased LOS in hospital was also much longer in CR-PA than that in non-drug-resistant PA. There was no significant difference in extra LOS between resistant bacteria and non-resistant bacterial infections in SA, SE, and KP.

In the process of medical treatment, medical staff have paid more and more attention to the impact of MDRO [21–23]. Whether MDRO infection can increase the length of stay in the hospital varies from study to study. Barrasa-Villar JI et al. [24] thought that hospital infections caused by MDROs did not appear to influence LOS compared with those produced by susceptible strains. However, the extra LOS due to a single MDRO in a specific type of infection was identified in other research studies [7–11].

In our study, the HAIs caused by some MDROs did not lead to longer LOS than those caused by susceptible strains. In some other studies, little or no effects of MDRO on the extended LOS were estimated both in ICU patients [25] and throughout the whole hospital [26]. There are many factors that can lead to longer LOS, such as patients with more comorbidities, patients in serious condition, or ICU stay [27]. Some vulnerable patients with infections need increased care whether they are caused by drug-resistant or susceptible microorganisms that results in prolonged LOS [28].

For hospitals, HAI will lead to the prolongation of average hospital stay which will reduce the number of patients admitted and reduce the hospital's medical income. In addition, in the first 13-18 days after admission, which are the efficient hospitalization days, the hospitalization cost is high, but after 18 days the hospitalization cost is lower than the average hospitalization cost [29]; therefore the treatment of new patients can bring more benefits to the hospital. Studies have found that HAIs occur during hospitalization with an average length of stay being 11 days [18] and that if these infections are controlled, then more patients can be treated.

Increased LOS can lead to more healthcare costs but calculating these costs is complicated due to time-dependent bias [30, 31]. Meredith L. Kilgore et al. [32] thought that it is possible that HAIs may lead to prolonged hospital stay which in turn increases the risk of infection (which was called mixing or endogeneity). In recent years, a number of studies have started to use tendentiousness scores [33] and tool variables [34] to correct this endogeneity but were not very successful.

The majority of articles used time-fixed methods (75%) [16]. Studies using time-fixed methods overestimate additional LOS attributable to HAI. Population heterogeneity, different case definitions, and different microorganisms lead to incomparability of different studies. People have been exploring different research methods, but they still mainly focus on time-fixed research.

One study [35] estimated the excess LOS attributable to HAIs, in which total LOS of patients with and without HAIs is overestimated because of failure to account for the timing of infection. In this study, the differences between the time-fixed and time-varying methods are fully discussed. They showed that the LOS due to HAI in studies using time-fixed method was 9.4 or 12.6 days longer on average than those using time-varying method. LOS due to HAIs is quite different according to the used methods. Overestimation of extended hospital stay may lead to incorrect assumptions of the effect of HAI prevention measures.

This study has two main limitations. First, our case-control matching principles do not take into account comorbidity and severity, which may lead to inaccurate assessment of the impact of HAIs on LOS. Second, we used the time-fixed method which could bias the effect of HAIs [36].

## 5. Conclusions

HAI can significantly increase the LOS. The increase varies according to hospital level, region, site of infection, and infected pathogen, and it also varies if the pathogens were multidrug-resistant. The HAI caused an increase in stay of 10.4 days. There was no significant difference in the increased LOS between different hospital levels. Comparison between regions shows that the increased LOS due to HAI in South China is obviously longer than other regions except the Northeast. The increased LOS of GI was significantly shorter than that of other sites.* E. coli* infection caused significantly shorter LOS. Comparison between different MDRO infections revealed that the increased LOS due to HAI caused by CR-PA is obviously longer than other MDRO infections.

## Figures and Tables

**Table 1:** The LOS due to HAI in different hospital levels (days).

Level	n	Case group		Control group		Increased LOS	t	*P*
		$\overset{-}{x}$	SD	$\overset{-}{x}$	SD			
Provincial or ministerial	627	23.2	1.8	12.3	1.9	10.9	24.89	<0.01
Prefectural or municipal	932	21.3	1.9	10.9	1.9	10.4	30.95	<0.01
District or county	560	19.3	2.1	9.6	1.9	9.7	21.94	<0.01
Total	2119	21.3	1.9	10.9	1.9	10.4	45.13	<0.01

**Table 2:** The LOS due to HAI in different regions.

Regions	n	Case group		Control group		Increased LOS	t	*P*
		$\overset{-}{x}$	SD	$\overset{-}{x}$	SD			
South	226	22.9	2.3	10.3	2.1	12.6	16.24	<0.01
Northeast	117	22.2	2.1	10.1	1.9	12.1	10.22	<0.01
North	501	23.3	1.8	11.9	1.8	11.4	23.25	<0.01
East	720	20.1	1.9	10.2	1.9	9.9	26.61	<0.01
Centre	209	20.6	2.0	11.0	1.9	9.6	14.84	<0.01
Southwest	148	20.5	2.0	11.8	1.9	8.7	9.28	<0.01
Northwest	198	19.9	1.8	11.7	1.8	8.2	12.68	<0.01
Total	2119	21.3	1.9	10.9	1.9	10.4	45.13	<0.01

**Table 3:** The LOS due to HAI in different sites.

Infection sites	n	Case group		Control group		Increased LOS	t	*P*
		$\overset{-}{x}$	SD	$\overset{-}{x}$	SD			
BSI	321	25.4	1.9	12.5	1.9	12.8	19.24	<0.01
SSI	405	23.4	1.9	11.5	1.8	11.8	22.77	<0.01
LRTI	537	22.3	1.9	11.1	1.9	11.2	21.71	<0.01
UTI	480	21.2	2.0	10.9	1.8	10.3	20.23	<0.01
GI	376	15.5	1.9	8.8	1.8	6.7	17.84	<0.01
Total	2119	21.3	1.9	10.9	1.9	10.4	45.13	<0.01

**Table 4:** The LOS due to HAI caused by different susceptible pathogens.

Pathogens	n	Case group		Control group		Increased LOS	t	*P*
		$\overset{-}{x}$	SD	$\overset{-}{x}$	SD			
KP	47	25.8	2.0	10.2	2.0	15.5	7.34	<0.01
EC	53	25.2	2.0	11.3	1.8	14.0	8.80	<0.01
SA	56	24.4	1.9	10.5	1.8	13.9	9.57	<0.01
PA	34	24.4	1.6	11.8	1.9	12.5	6.66	<0.01
AB	16	27.8	1.9	15.6	1.8	12.2	4.38	<0.01
SE	29	21.5	1.9	10.1	2.0	11.4	5.54	<0.01
E. coli	130	21.0	1.8	11.2	1.7	9.8	11.27	<0.01
Total	365	23.3	1.9	11.1	1.8	12.2	20.73	<0.01

**Table 5:** The LOS due to HAI caused by different MDROs.

MDRO	n	Case group		Control group		Increased LOS	t	*P*
		$\overset{-}{x}$	SD	$\overset{-}{x}$	SD			
CR-PA	31	38.8	2.4	12.2	2.0	26.5	6.50	<0.01
VRE	11	35.1	1.9	15.3	1.6	19.8	5.06	<0.01
CR-E. coli	28	27.5	1.9	10.8	1.8	16.8	7.43	<0.01
CR-AB	49	29.4	1.9	13.6	1.9	15.7	6.79	<0.01
CR-KP	26	26.8	1.7	12.6	2.1	14.3	5.75	<0.01
MRSA	60	27.6	1.8	14.3	1.8	13.3	7.29	<0.01
ESBL E. coli	124	22.8	1.9	11.1	1.9	11.7	12.62	<0.01
ESBL KP	38	24.5	1.9	13.2	1.8	11.3	5.40	<0.01
MRSE	18	22.9	2.1	13.2	1.9	9.7	3.19	0.01
Total	381	26.4	2.0	12.4	1.9	14.0	20.36	<0.01

## Data Availability

The data used to support the findings of this study are included within the article.
